# Avid ^18^F-FDG uptake of pectoralis major muscle: an equivocal sequela of strenuous physical exercise

**DOI:** 10.2349/biij.5.2.e7

**Published:** 2009-04-01

**Authors:** F Fathinul, WFE Lau

**Affiliations:** 1 Department of Radiology, Heart, Stroke and Cancer Centre, Kuala Lumpur, Malaysia; 2 Centre for Molecular Imaging, The Peter Mac-Callum Cancer Centre, Melbourne, Australia; 3 University of Melbourne, Melbourne, Australia

**Keywords:** ^18^F-FDG uptake, pectoralis major muscle, exercise

## Abstract

Avid functional ^18^F-FDG uptake of skeletal muscle is a known false positive finding of PET-CT study especially after involuntary muscle exercise just prior to the study. We describe the case of a 50-year-old man in whom the finding of avid ^18^F-FDG uptake of pectoralis major muscle was encountered during investigation of metastatic melanoma.

## INTRODUCTION

The purpose of this case report is to highlight an unexpected pitfall of ^18^F-FDG uptake of pectoralis major muscle, which is potentially caused by exercise of the upper limb muscles.

^18^F-FDG is a common PET radiotracer used in oncology and its property as a glucose analogue is known to play a vital role in the detection of most malignant tissue. Nevertheless, ^18^F-FDG is not a tumour-specific substance as it shows non-specific affinity in other normal tissue especially in the skeletal muscle [1].

Active skeletal muscle is a common area for interpretative pitfall which is related to physiological FDG uptake [1]. In addition, other reasons for ^18^F-FDG uptake in muscle are also observed in diabetic patient following administration of insulin, surgical intervention or following immunosuppressive therapy.

Muscle uptake can be attributed to voluntary or involuntary muscle activity. Voluntary muscle activity consists of activities such as talking, chewing, and exercising-induced rhabdomyolysis. Involuntary muscle activity would include laboured breathing or stress-induced muscle spasms.

Normal muscles accumulates little FDG, uptake of grade 1 or less. Muscles exercised just prior to or around the time of FDG injection can exhibit uptake of grade 3 to 4 [2]. Notwithstanding the known normal physiological FDG uptake in muscle, grade 3 to 4 muscle exercised within more than 24 hours remains a poorly defined issue.

## CASE REPORT

**Figure 1:**
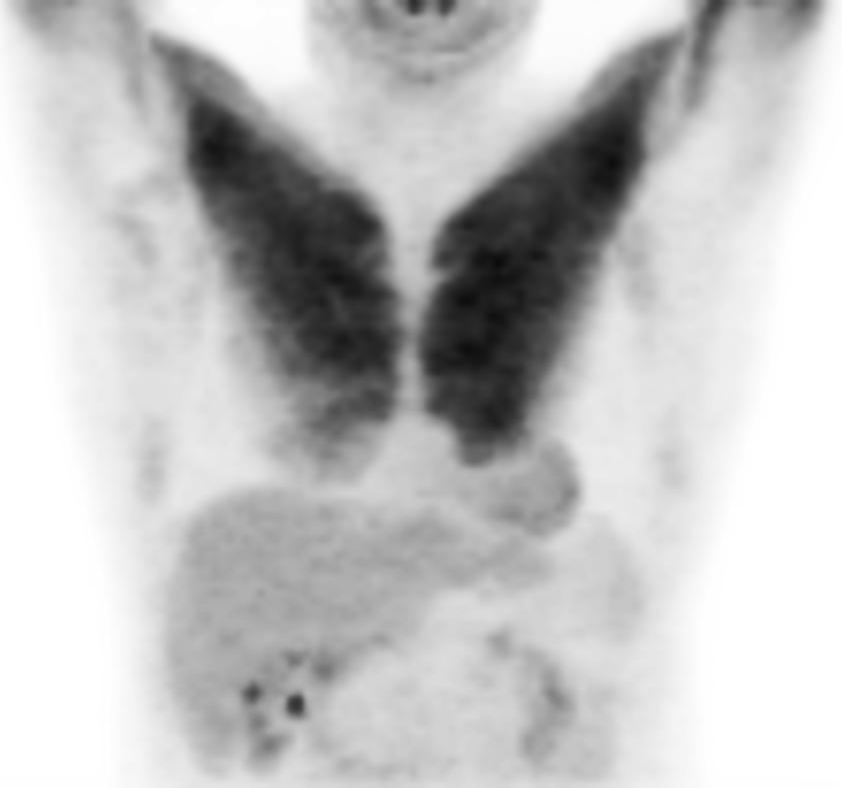
FDG PET scan (coronal view. Grade 3 to 4 symmetrical F-18 FDG uptake of the pectoralis major muscle) [Image courtesy of Centre for Molecular Imaging, Peter MacCallum Cancer Centre, East Melbourne, Victoria, Australia].

A 50-year-old gentleman with a small right ankle melanoma had been successfully managed with surgical removal of the melanoma in 2001. He had right inguinal nodal recurrence after 6 years disease-free progression. A PET-CT study performed in June 2007 revealed unexpected intense symmetrical uptake of the pectoralis major muscle (Figure 1). The patient was noted to have undergone strenuous upper limbs exercise 24 hours prior to the imaging study. Clinically, the disease showed a favourable response following several courses of high dose adjuvant interferon alfa- 2b therapy. Biochemical blood analysis was generally unremarkable aside from minimal derangement of the liver function.

## DISCUSSION

In our case, an avid ^18^F-FDG uptake in pectoralis major muscle is conceptually attributed to some form of strenuous eccentric exercise 24 hours prior to PET-CT study.

Exercise is a well known mechanism that is capable of inducing various components of the immune system [3]. IL-6 is a lymphokine that would substantiate immune-protection against muscle damage by maintaining glucose uptake in the muscle following prolonged exercise [4]. Glucose uptake by skeletal tissue is insulin-dependent via recruitment of the GLUT4 (glucose transporter) from the interior of the cell to the plasma membrane. In human skeletal muscle, exercise increases GLUT-4 and hexokinase II and glycogenin gene expression. Nevertheless, in-vivo study reveals that one hour of moderate intensity exercise increases Hexokinase II transcription mRNA and protein levels up to 3 hours after the end of exercise [4].

The fact is that a substantiated FDG accumulation in skeletal muscle as shown in our case cannot be entirely explained by the influence of intramuscular glucose uptake for more than 24 hours following exercise.

A possible explanation of what appears to augment FDG avidity in this case might have been attributed to ongoing rhabdomyolysis following strenuous exercise. Rhabdomyolysis appears to be a relatively common sequela of strenuous exercise [4]. Of note, numerous case reports have linked rhabdomyolysis to strenuous activities such as military basic training and weight lifting [5]. This is further substantiated by a large screening to date involving 337 military recruits whose blood were sampled during their first six days of conditioning which revealed that an approximately 40% of the subjects have some degree of rhabdomyolysis [5].

It is noteworthy that the cause of prolonged avid FDG muscle uptake in our case remains unclear as there were neither symptoms related to muscle damage i.e aching nor available biochemical analysis pertaining to the level of creatinine kinase and urine myoglobin levels.

## CONCLUSION

Unexpected FDG muscle uptake may occur more than 24 hours following exercise as illustrated in our case. The exact explanation is unclear but underlies the importance of adequate patient preparation and instructions before a FDG PET scan.
